# Metabolic and Epigenetic Regulation by Estrogen in Adipocytes

**DOI:** 10.3389/fendo.2022.828780

**Published:** 2022-02-22

**Authors:** Jan-Inge Bjune, Pouda Panahandeh Strømland, Regine Åsen Jersin, Gunnar Mellgren, Simon Nitter Dankel

**Affiliations:** ^1^ Hormone Laboratory, Department of Biochemistry and Pharmacology, Haukeland University Hospital, Bergen, Norway; ^2^ Mohn Nutrition Research Laboratory, Department of Clinical Science, University of Bergen, Bergen, Norway

**Keywords:** sexual dimorphism, steroids, estrogen, adipocyte, epigenetic

## Abstract

Sex hormones contribute to differences between males and females in body fat distribution and associated disease risk. Higher concentrations of estrogens are associated with a more gynoid body shape and with more fat storage on hips and thighs rather than in visceral depots. Estrogen-mediated protection against visceral adiposity is shown in post-menopausal women with lower levels of estrogens and the reduction in central body fat observed after treatment with hormone-replacement therapy. Estrogen exerts its physiological effects *via* the estrogen receptors (ERα, ERβ and GPR30) in target cells, including adipocytes. Studies in mice indicate that estrogen protects against adipose inflammation and fibrosis also before the onset of obesity. The mechanisms involved in estrogen-dependent body fat distribution are incompletely understood, but involve, e.g., increased mTOR signaling and suppression of autophagy and adipogenesis/lipid storage. Estrogen plays a key role in epigenetic regulation of adipogenic genes by interacting with enzymes that remodel DNA methylation and histone tail post-translational modifications. However, more studies are needed to map the differential epigenetic effects of ER in different adipocyte subtypes, including those in subcutaneous and visceral adipose tissues. We here review recent discoveries of ER-mediated transcriptional and epigenetic regulation in adipocytes, which may explain sexual dimorphisms in body fat distribution and obesity-related disease risk.

## Introduction

Sexual dimorphism in obesity and related cardiometabolic risk involves differences in fat distribution (1, 2), described by Vague already in 1947 (3). Most body fat is stored in two main white adipose tissue (WAT) depots; subcutaneous adipose tissue (SAT) and visceral adipose tissue (VAT). Increased visceral adiposity is particularly associated with increased mortality and risk of a range of metabolic conditions including insulin resistance, type 2 diabetes, and cardiovascular disease (4–11). In contrast, preferential fat accumulation on the hips, thighs and other subcutaneous sites in females compared to males may help explain the lower risk of metabolic diseases generally seen in females (11). VAT (omental and mesenteric fat) normally constitutes about 10-20% of total body fat in males and 5-10% in females (12), although these percentages vary greatly for different individuals (4–6, 8, 13). There is a relative increase in adipose tissue (AT) mass and decrease in muscle mass with age (14, 15), which is associated with altered concentrations and activity of sex hormones (16), including testosterone and estrogens, which are potent regulators of adipogenesis and energy metabolism (17, 18). Importantly, with loss of estrogens after menopause, females often begin storing more VAT and have higher risk of metabolic diseases, more like males (1, 19, 20). This shift in AT function and distribution can in turn alter the metabolic functions of other tissues, in part *via* changes in adipokine secretion, release of lipids for energy expenditure or storage in tissues such as liver, muscle and heart, and other mechanisms (2, 21).

Among all natural or synthetic estrogens (22), endogenous estrogens in humans consist of estrone (E1), estriol (E3) and 17β-estradiol (E2), the latter being the most biologically active (22, 23). In premenopausal women, E2 is the dominating estrogen, while E1 produced by adipose tissue is more important after menopause (24). Androgens are converted to estrogens by the enzyme aromatase, thus linking the sex hormones in both males and females (25). Estrogens bind to two ‘classical’ estrogen receptor (ER) subtypes, alpha (ERα) and beta (ERβ), which have multiple isoforms and exhibit distinct tissue expression patterns (26). E2 has similar affinity to both receptors (26, 27). Estrogen-mediated activation of ER-dependent transcriptional activity alters epigenetic programming and global gene expression patterns, contributing critically to the cellular effects of estrogens, such as in breast cancer (28) and hippocampal memory formation (29). Thus, in breast cancer cells, estrogen deprivation has been found to cause DNA hypermethylation and histone deacetylation and consequent downregulation of global gene expression, which was largely reversed by E2 re-stimulation (30). It is possible that such epigenetic mechanisms are central in ER subtype-specific effects, given tissue differences in ER subtype expression levels (26).

Studies on mechanisms of sexual dimorphism in body fat distribution have pointed to the role of sex hormones as well as the microenvironment and cell-specific properties within fat depots (31). Due to the importance of epigenetic/transcriptional programming for the unique functional properties of different adipocyte subtypes (32), it may be critical to determine how and to what extent estrogens contribute to these distinct properties, and consequently to sex differences in body fat distribution and associated risk of metabolic diseases. In this review, we discuss the role of estrogens in adipose tissue distribution and function, and emphasize emerging knowledge of estrogen-dependent epigenetic mechanisms that may govern sexual dimorphism in obesity and adipogenesis.

## Role of Estrogen in Adipose Tissue

**Figure 1:**
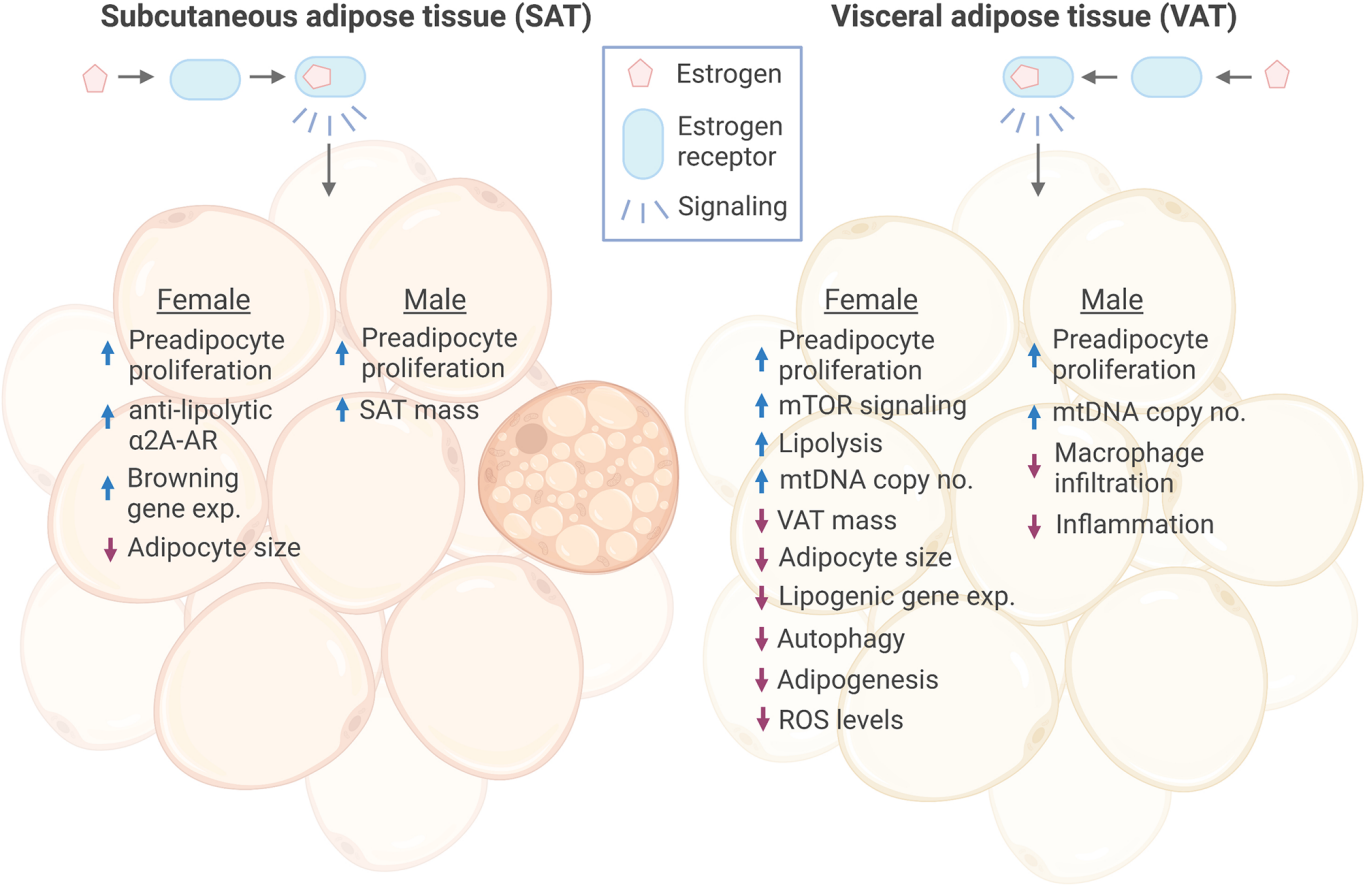
Effects of estrogen signaling in female and male SAT and VAT. Estrogen signaling in subcutaneous adipose tissue (SAT) and visceral adipose tissue (VAT) of both sexes has been found to promote increased proliferation of preadipocytes. Estrogen has been shown to promote anti-lipolytic effects through increasing the expression of a2A-AR in female SAT, which may, at least in part, explain the concomitant increase in SAT mass and overall anti-obesogenic effect of estrogen. In addition, Estrogen induced expression of several browning genes in female SAT. In response to estrogen, female VAT showed increased lipolysis, while lipogenic gene expression was decreased, together resulting in reduced VAT mass. On the contrary, estrogen increased male SAT volume. Adipocyte size was reduced in both female SAT and VAT by estrogen, while there were no reports of this in males. However, estrogen decreased macrophage infiltration and inflammation in male VAT. Female VAT has been shown to have reduced autophagy, adipogenesis and ROS levels in response to estrogen treatment. ER, estrogen receptor; a2A-AR, alpha2A-adrenergic receptors; exp., expression. Figure created in BioRender.com.

ERα expression is inversely associated with obesity in both females (33), males (34) and over 100 different strains of inbred mice (34). In human (20) as well as rodent (35) females, the decline in circulating E2 after menopause corresponds to increased fat mass and lower glucose tolerance. Conversely, estrogen replacement therapy reverses these effects (35–38). Moreover, E2 treatment in nutritionally challenged female mice reduced VAT mass and adipocyte size, and altered gene expression of lipogenic markers, adipokines, specific nuclear receptors, and thermogenic markers (39). However, effects of estrogen-ERα signaling often differ greatly, both between the two sexes, and between SAT and VAT (as described in detail in the sections below and summarized in **Figure 1**).

Estrogen exerts anti-obesity effects through multiple mechanisms, such as central regulation of energy intake and expenditure (reviewed in (40)). However, estrogen also has direct effects in WAT, and it has been shown that loss of estrogens has a much stronger effect on gene expression in WAT compared to for example the hypothalamus (41). In WAT, E2 is shown to decrease expression of genes involved in triglyceride synthesis (lipogenesis) and promote catecholamine-induced lipolysis (42, 43). While estrogens can affect adiposity, adiposity can also increase the production of estrogens locally in several tissues. Although AT is not steroidogenic, it is the most important site for steroid production outside the gonads due to the presence of the aromatase cytochrome P450 enzyme, which converts androgens taken up from the circulation into estrogens (44, 45). Due to the presence of aromatase in AT, the locally produced estrogen can affect metabolism independent of plasma E2 levels (44). In rats, it has been shown that local E2 levels are about tenfold higher in AT compared to the circulation (44). Conversely, another study observed no statistically significant differences between the sexes in neither plasma nor overall adipose E2 levels between male and female rats, while a significant depot-dependent effect was found in both sexes, where E2 levels showed 1.5-2-fold higher levels in SAT compared to different VAT depots (44).

Female ovariectomized mice display increased VAT and reduced leptin sensitivity compared to controls, which upon E2 administration can be restored to levels seen in intact cycling females (46). Interestingly, male mice given E2 show decreased insulin sensitivity, increased SAT volume, higher sensitivity to leptin, and overall increased body fat (46), at least in part explained by reduced physical activity and energy expenditure (46). No stimulatory effects on food intake were seen, and estrogen may rather have leptin mimetic/anorectic functions that suppress food uptake (46, 47), pointing to other tissue-specific obesogenic effects of E2 in males.

In male humans, an increase in AT mass is associated with increased levels of aromatase (48, 49), and hence increased ability to synthesize estrogens (50). Conversely, administration of aromatase inhibitors increases the testosterone-estrogen ratio and reverses hypogonadal obesity, resulting in the stimulation of muscle protein synthesis and increased muscle mass (51, 52). The aromatization process progressively reduces testosterone levels and elevates estrogen levels in males (52). Decreased testosterone concentrations in males are associated with elevated concentrations of leptin, which is produced by fat cells as a reflection of fat stores. Further expansion of visceral AT and production of aromatase through this hypogonadal-obesity cycle may result in a vicious cycle of continued visceral AT expansion and insulin resistance (52). On the other hand, higher levels of AT aromatase activity in male mice leads to a decreased adipose tissue inflammation and improved insulin sensitivity (53). Given the generally protective effect of estrogen against visceral adiposity, an important question is whether estrogen has different effects in males compared to females. To answer this question, we need more detailed insight into how estrogen exerts its biological effects, and whether there are differences in intracellular signaling mechanisms in relevant metabolic cells between the sexes.

## Mechanisms of Action and Metabolic Regulation by Estrogen in Adipose Tissue

In the early 1990s, Mizutani et al. and Pedersen et al. reported the presence of ER along with other steroid receptors such as glucocorticoid and androgen receptors, but not progesterone receptors, in human mature adipocytes (54, 55). The effect of estrogen on AT distribution is mainly controlled by the adipocyte ERα (56), and the estrogen-ERα signaling has anti-obesity effects (57). In a rat study by Rodriguez-Cuenca et al., VAT from both males and females exhibited lower levels of E2, but higher expression levels of ERα and ERβ compared to SAT (44). These data suggest that VAT is more sensitive to E2 than SAT (44), which supports the observation that estrogen-stimulated lipolysis occurs mainly in visceral compartments (58). Moreover, it may explain why ERα-KO mice of both sexes gain weight only in visceral compartments (56).

Estrogen has been shown to reduce adipogenesis through activation of mTOR signaling, promoting inhibition of PPARγ (40, 59–62) or reduction of autophagy in female VAT (63). Importantly, the pro-lipolytic effect of E2 has been found to be blunted specifically in female SAT (64), *via* estrogen-mediated increase in anti-lipolytic α2A-adrenergic receptors (59, 64). Interestingly, this was not observed in VAT (64) which may help to explain why only SAT and not VAT in females is affected by changes in serum levels of estrogen and how estrogen overall has anti-obesity effects but at the same time promotes fat storage subcutaneously (59, 64). These effects of estrogen may explain some of the findings in genome-wide association studies with more than 224,000 individuals (65), showing that metabolic changes are likely involved in the sexual dimorphism of obesity and fat distribution, implicating mechanisms *via* differential control of adipogenesis and insulin resistance between sexes (1, 65, 66).

Studies have previously shown that estrogen and its receptors are involved in regulating preadipocyte and adipocyte growth and function, and some differences between the sexes are reported (67–69). Interestingly, E2 stimulates the proliferation of preadipocytes from both sexes (67). However, subcutaneous and visceral preadipocytes from females were more responsive to E2 and proliferated faster compared to preadipocytes from males (67). Both male and female mice harboring a knockout (KO) of ERα showed increased levels of body fat compared to their wild-type (WT) littermates, despite similar body weights (68). The same study reported that these male and female ERα KO mice had larger adipocytes, and higher expression of markers of macrophage infiltration and markers of fibrosis than WT mice (68). Another report found that female whole body ERα KO mice also showed reduced adiponectin expression, and increased fibrosis and inflammation (69).

Furthermore, similar phenotypes were observed in both male and female adipocyte-specific ERα knockout (AdipoERα) mice compared to whole-body KOs, with some exceptions (69). Despite no increase in weight gain, the male AdipoERα mice showed reduced glucose clearance as measured by an oral glucose tolerance test, suggesting adipocyte dysfunction in the absence of estrogen-ERα signaling in males. Surprisingly, glucose clearance in female AdipoERα mice, showing increased weight gain compared to WTs, was not affected (69). While both male and female AdipoERα mice showed increased adipocyte size compared to their WT counterparts, only adipocytes of male mice had increased expression of markers of macrophage infiltration, inflammation and fibrosis, indicating sex-dependent regulation of adipocyte function (68). Interestingly, adipocyte-specific loss of ERα in ERβ deficient mice leads to lower glucose tolerance also in female mice (as seen for male AdipoERα mice with expression of ERβ), as well as increased markers of inflammation and fibrosis. These findings suggest that ERβ may regulate glucose homeostasis, fibrosis and inflammation in female AdipoERα mice but not in males (68).

Moreover, E2, *via* ERβ signaling, increased the expression of thermogenic uncoupling protein-1 (UCP-1) in mouse brown adipose tissue (BAT), leading to increased energy expenditure and thus reduced fat mass (60). In 2018, it was shown that activation of ERs in white adipocytes in both humans and mice increased markers of beiging (70). However, whether there are sex differences in this regulation remains to be determined. Of note, both the anorectic function of E2 as well as its role in increasing the energy expenditure can also be mediated through both ERα and β in the hypothalamic area of the brain (71).

Estrogen signaling also is best known to affect gene expression in target tissues, but can also affect processes outside the nucleus, involving ion channels and protein kinases, which is so-called non-genomic or non-nuclear signaling. In contrast to the relatively slow activation of gene transcription, these non-genomic pathways occur rapidly (within seconds or minutes) *via* membrane-associated forms of the ERs (72). It has been shown that E2 treatment of ovariectomized mice rapidly increased fat oxidation through activation of AMPK (42). Moreover, E2 can inhibit glucose oxidation in adipocytes through non-genomic mechanisms (73). Estrogen may also bind other non-classical receptors, including GPR30, which is a G protein-coupled estrogen receptor (GPER) in the endoplasmic reticulum that has a high affinity for E2 (74, 75). These pathways have been mostly studied in neurons or pancreatic β cells, and have been suggested to be the most important mediators of estrogen signaling in these tissues (40, 76). However, recent *in vitro* and *in vivo* studies have shown that GPR30 plays an important role in adipogenesis by reducing the fat mass and adipocyte size (77). Compared to BAT, GPR30 is highly expressed in WAT (77). Deletion of GPR30 by reducing plasma insulin and leptin levels protects female mice from developing obesity, glucose intolerance and insulin resistance after nutritional challenge (77). How GPR30-mediated estrogen signaling interacts with mechanisms of epigenomic regulation remains to be determined.

### Adipose Tissue Gene Regulation by Estrogen Receptors

ERs can bind directly or indirectly to promoters of target genes to repress or activate their expression (26). Manipulation of estrogen levels or ERs have provided insights into adipocyte target genes and thereby the mechanisms of ER-mediated gene regulation. For example, loss of estrogens by ovariectomizing reduced WAT expression of *glutathione peroxidase 3* (*Gpx3)* (41), a gene important for the protection of cells from oxidative stress in the form of reactive oxygen species (ROS) (78). Furthermore, E2 reduced ROS levels and enhanced browning in female mouse SAT through promoting macrophage heme *oxygenase-1* (*Hmox1*, also known as *HO-1*) expression (79). Similarly, E2 treatment of 3T3-L1 adipocytes increased expression of genes encoding the ROS reducing antioxidants HO-1, NAD(P)H:quinone oxidoreductase 1 (*NQO1*) and glutamate-cysteine ligase (*GCL*), directly in the adipocytes (80). High levels of ROS have previously been linked to decreased mitochondrial respiration (81) and increased fat storage (82), which are typical hallmarks of adipocyte dysfunction (83). Correspondingly, postmenopausal females showed increased VAT ROS levels compared to premenopausal individuals (80). Together, these data may suggest that intact E2 signaling could, through regulation of genes involved in antioxidant processes, play a role in increasing the resilience to nutritional/metabolic stress and prevent adipose dysfunction, a key contributor of obesity and metabolic syndrome (84).

In support of this theory, adipose-specific deletion of *Estrogen receptor 1* (*Esr1*, gene encoding ERα) in both female and male mice have recently been shown to decrease mitochondrial DNA (mtDNA) copy number in both WAT and BAT (34) (**Figure 1**). The investigators demonstrated that ERα binds directly to the nuclear-encoded *mtDNA polymerase subunit γ (Polg1)*, thereby controlling mtDNA replication in WAT (34). Moreover, loss of ERα was further accompanied by reduced expression of key genes involved in mitochondrial biogenesis (*Pgc1b*, *Nrf1*), and transcription (*Polrmt*) (34). Other studies have previously shown that *NRF1* is under control of E2-mediated ERα and ERβ activities in other tissues such as breast cancer, mammary glands, and the uterus (85, 86). In female mice BAT, ERα is necessary for mitochondrial remodeling through *Dynamin-related protein 1* (*Drp1*) (34), and thermogenesis through *Ucp1* (34) and *Cidea* (41). Overall, these data suggest that estrogen signaling is important for maintaining mitochondrial function in females, an important prerequisite for preventing adipocyte dysfunction and metabolic complications (87).

A number of microRNAs (miRNAs) have been found to play crucial roles in both white and beige/brown adipocyte development and function (reviewed in (88)). Knockdown of ERα in rat bone marrow-derived mesenchymal stem cells (BMSCs) has been found to alter the expression of almost 200 miRNAs, including downregulation of miR-210-3p, accompanied with increased Pparg protein levels and reduced expression of the osteogenic regulator Runx2 (89). Conversely, overexpression of miR-210-3p was found to increase Wnt signaling and promoted osteogenesis over adipogenesis (89). Interestingly, endometriosis is an estrogen-driven inflammatory disease characterized by reduced BMI and abnormal levels of circulating miRNAs (90), including miR-342 (91) and Let-7b (92, 93). Overexpression or inhibition of these miRNAs in primary preadipocytes from healthy donors altered the expression of *C/ebpa*, *C/ebpb* and *Pparg* (94). Of note, miRNAs may affect gene expression not only in the cells they are produced, but also in distant cells and tissues through secreted extracellular vesicles, including exosomes (95). Importantly, small motifs in the miRNAs have recently been found to dictate their retention or secretion, with white adipocytes demonstrating by far the highest production and secretion rates per cell compared to several other cell types (96). Thus, future studies should be better equipped to predict and assess local and systemic effects of ER-regulated miRNAs.

## Estrogen-Mediated Epigenetic Regulation in Adipocytes

**Figure 2:**
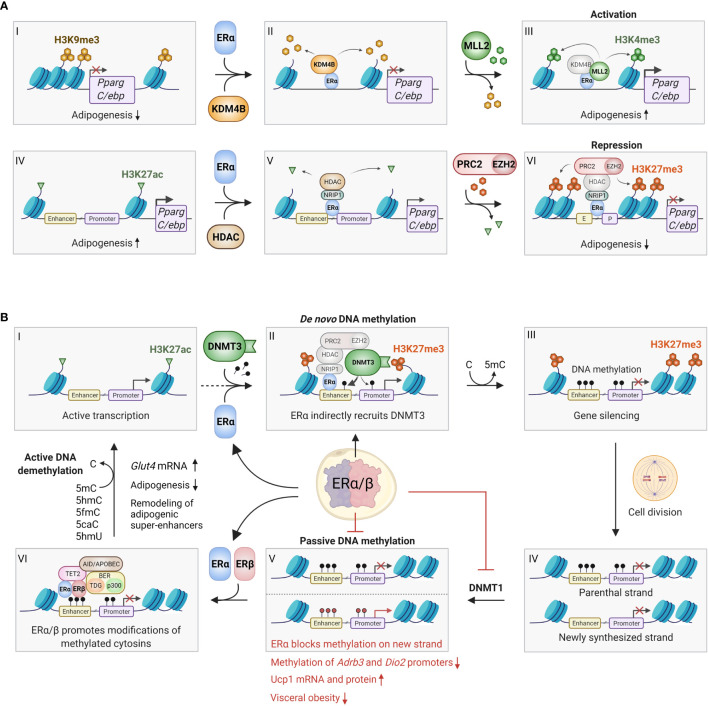
Epigenetic effects of ERα/β in adipocytes. **(A)** ERα can bind to promoter regions with repressive H3K9me3 marks (I-VI), where it recruits the histone demethylase KDM4B (also known as JMJD2B), which specifically removes these marks (II). This enables the recruitment and activity of the methyl transferase MLL2, which trimethylates lysine 4 on histone 3, forming activating H3K4me3 marks, which promotes gene expression (III). This process may occur on the promoters of *Pparg* and *C/ebp*, promoting adipogenesis. Conversely, ERα can also bind to actively transcribed genes characterized by H3K27ac marks (IV), where it binds various coregulators, including NRIP1, that enables binding of histone deacetylases (HDACs), which remove the acetyl groups on H3K27 (V). Finally, the ERα/NRIP1/HDAC complex can further bind the PRC2/EZH2 polycomb complex, which adds methyl groups to form repressive H3K27me3 marks (VI). This process can also occur on the *Pparg* and *C/ebp* promoter/enhancers, inhibiting adipogenesis. Although the repressive pathway appears most predominant, further studies should investigate whether the activating pathway indeed plays a role in certain preadipocyte/mesenchymal stem cell subpopulations. **(B)** ERα and ERβ affects DNA methylation through several mechanisms. ERα promotes *de novo* methylation and gene silencing by binding to actively transcribed regions (I), where the ERα/HDAC/PRC2/EZH2 complex first converts activating H3K27ac marks to repressive H3K27me3 marks (see **Figure 2A** IV-VI for details). The DNA methyl transferase DNMT3 recognizes the H3K27me3 marks, and stabilized by the ERα/HDAC/PRC2/EZH2 complex it adds a methyl group to cytosine residues on the surrounding DNA, leading to stable gene silencing (II-III). Conversely, ERα can inhibit passive DNA methylation after cell division. This occurs by transcriptional inhibition of DNMT1, which copies the DNA methylation pattern of the old DNA strand onto the newly synthesized DNA (IV-V). Red methyl groups (bottom panel V) represent hypomethylated regions in response to ERα-mediated repression of DNMT1, leading to increased beiging. ERα and/or ERβ can also promote active demethylation by recruitment of TET2, AID/APOBEC/BER complexes, which alter methylated cytosines in numerous ways that ultimately restores unmodified cytosine (VI-I). Active demethylation likely remodels adipogenic super-enhancers, and has been found to inhibit adipogenesis and increase Glut4 expression. C, Cytosine; 5mC, 5-methylcytosine; 5hmC, 5-hydroxymethylcytosine; 5fmC, 5-formylcytosine; 5caC, 5-carboxylcytosine; 5hmU, 5-hydroxymethyluracyl. Figure created in BioRender.com.

Epigenetics plays a causal role in the development of obesity (97), and adipogenesis is extensively regulated by DNA methylation and demethylation, histone tail modifications and chromatin remodeling (97, 98). Strikingly, E2-bound ERs have been shown to be involved in these epigenetic processes in various tissues through recruitment of co-regulators and epigenetic remodeling enzymes (99, 100). We will here review general known mechanisms of epigenetic regulation *via* estrogens and highlight known aspects in adipocytes (**Figure 2**).

### Histone Modifications by ERα

Cellular DNA is wrapped around histone proteins to form nucleosomes and higher-order chromatin structures (101, 102), which constitutes a major layer in transcriptional regulation (101, 102). H2, H3 and H4 histone family members, with tails of various lengths, are subjected to extensive post-translational modifications, including methylation and acetylation (101, 102). ERα interacts with, and promotes the activity of MLL2 (99, 103), a specific H3K4 histone methyltransferase (HMT) that confers epigenetic activation of gene expression (104) (**Figure 2A**). Mutations in MLL2 lead to insulin resistance and reduced glucose tolerance in mice (105) and humans (98, 106). Because MLL2-dependent H3K4me3 activating marks are mutually exclusive with repressing H3K9me3 marks, the MLL2/ERα complex also includes KDM4B, a H3K9 demethylase that coordinates the conversion from repressive to activating marks (103). In preadipocytes, KDM4B is known to act on the promoters of *Pparg* and *C/ebp* and promote adipogenesis (107). Taken together, ERα may promote adipogenesis through KDM4B/MLL2 (**Figure 2A**), but this remains to be confirmed. Of particular interest would be whether this mechanism exists predominantly in subcutaneous (gluteal and femoral) WAT.

In contrast, as detailed further above, estrogen and/or ERα has mainly been found to *inhibit* adipogenesis (108–111). ERα mediates epigenetic silencing by recruiting histone deacetylase HDAC1 and HMTs like EZH2 to convert activating H3K27ac marks to repressive H3K27me3 marks (99) (**Figure 2A**). In rats, E2 treatment increased the binding of ERα/EZH2 to the promoters of *Pparg*, C/ebpa and *Cfd* (encoding Adipsin) in mesenchymal stem cells (MSCs), leading to increased H3K27 methylation and repression of these genes (112). These data support a predominantly inhibitory effect of estrogen on adipogenesis, and this effect is at least partly due to epigenetic silencing of adipogenic master regulators.

### DNA Methylation and Demethylation by ERα

DNA methylation on CpG islands (99), which are present in most promoters (113), has a repressive effect on gene expression (114). This reaction can be catalyzed by two types of DNA methyltransferases (DNMTs) depending on the purpose of the methylation. While DNMT1 is active during cell division where it copies the parental DNA methylation pattern, DNMT3 can establish new methylation patterns, also known as *de novo* DNA methylation (115). ERα promotes the latter by indirect recruitment and activation of DNMT3 (99) to EREs (**Figure 2B**). Thus, mapping the genomic binding pattern of ERα in different adipose tissues at different developmental stages is critical to understand its epigenetic effects. Strikingly, ERα has shown a strong preference for binding to intergenic regions (116). Interestingly, about half of the CpG islands are also found in intergenic regions, and have recently been shown to be an essential part of poised enhancers, acting as anchors between the enhancer and target promoters (113). Consequently, methylation of CpG islands plays a crucial role in determining enhancer-promoter selectivity. Importantly, there are significant changes in enhancer interactions during adipocyte differentiation (117). Collectively, ERα may be involved in methylation-dependent regulation of enhancer-promoter interactions during adipogenesis. However, future studies are needed to test this hypothesis.

ERα and ERβ are also involved in *de*methylation, both passively during cell division by transcriptional inhibition of DNMT1, and actively by interacting with a range of enzymes that modify and remove the methyl group (99, 118) (**Figure 2B**). ERβ promotes active demethylation and increased expression of *Glut4* in mouse embryonic fibroblasts by recruiting the demethylation machinery to the *Glut4* promoter (119, 120). This ER-bound demethylation machinery includes TET2, which has been shown to inhibit adipogenesis (121), and p300, a known master epigenetic writer of enhancers during adipogenesis (122). It is therefore plausible that ERα and/or ERβ-dependent DNA demethylation is involved in the epigenetic regulation of adipogenesis, although this was not directly investigated. However, E2 has been shown to epigenetically promote beiging in mice by promoting demethylation of the *Adrb3* and *Dio2* promoters, leading to increased *Ucp1* expression (39). These changes were accompanied by reduced visceral lipogenic gene expression, improved fatty acid utilization, which reversed diet-induced visceral obesity and glucose intolerance (39). Moreover, activation of both ERα (70) and ERβ (123) has been shown by others to activate WAT browning (124). Taken together, ERα may promote thermogenesis by relieving repressive methylation marks on key positive regulators of beiging and mitochondrial uncoupling.

## Discussion

At the time morphological differences between individuals with obesity were first described (3), the direct influence of sex hormones on adipocytes had not been explored. Since then, much has been learned about how metabolic processes differ by sex and how estrogen affects developmental, metabolic and epigenetic processes, including adipogenesis and the fate of adipocyte progenitor cells towards thermogenic brown/beige or white fat cells.

In the research performed by Pedersen et al., Santos et al. and Zhou et al. (34, 64, 70), an effort has been made to differentiate the mechanism of estrogen signaling in different subtypes of adipocytes. However, despite technological advances allowing improved distinction of the metabolic properties of subcutaneous and visceral adipose depots, the effects of estrogen on distinct subtypes of fat cells in different depots remains to be described. More detailed insight into the role of estrogen signaling in adipocyte subtypes may be critical, as different adipocytes possess unique metabolic and endocrine profiles regardless of adipogenic capacities (125, 126).

The first evidence for epigenetic control of adipogenesis by estrogen was provided by the study of Rüegg et al. in 2011 (119), and progress has since been made in this field of research. However, more research is needed to fully understand estrogen-dependent mechanisms in different adipose tissue depots and adipocyte subtypes, and to what extent these mechanisms are distinct in males and females. New detailed insight into estrogen-mediated epigenetic changes may also help to assess health effects of environmental xenoestrogens, which partly act *via* epigenetic changes (127). Furthermore, it will be important to clarify functional differences and similarities between ERα and Erβ in metabolic and epigenetic regulation in different adipose cell types and depots.

At the same time, we must consider that estrogen effects on adipocytes are not limited to the classical types of ERs. For example, Wang et al. revealed that the relatively recently described non-genomic estrogen receptor GPR30 regulates adiposity in mice in a sex-specific manner (77). A relevant challenge is therefore also to evaluate whether GPR30-mediated estrogen signaling might interact with mechanisms of epigenomic regulation.

In conclusion, the emerging knowledge of estrogen-mediated metabolic and epigenetic regulation in different adipocytes provides a deeper understanding of how cellular programming regulates metabolic health. Further research in this area may uncover new molecular targets for improving body composition, insulin resistance and reducing the risk of lifestyle-related diseases.

## Author Contributions

Conceptualization and literature investigation: SD, PPS, J-IB, RÅJ, and GM. Original draft preparation: PPS, J-IB, RÅJ, and SD. Writing and editing: SD, J-IB, RÅJ, PPS, and GM. Figures preparation: RÅJ and J-IB. Supervision: SD and GM. All authors contributed to the article and approved the submitted version.

## Funding

This work was funded by grants received from the Trond Mohn Foundation (BFS2017NUTRITIONLAB) and the Western Norway Regional Health Authority (912010).

## Conflict of Interest

The authors declare that the research was conducted in the absence of any commercial or financial relationships that could be construed as a potential conflict of interest.

## Publisher’s Note

All claims expressed in this article are solely those of the authors and do not necessarily represent those of their affiliated organizations, or those of the publisher, the editors and the reviewers. Any product that may be evaluated in this article, or claim that may be made by its manufacturer, is not guaranteed or endorsed by the publisher.
